# The impact of medication belief on adherence to infliximab in patients with Crohn’s disease

**DOI:** 10.3389/fphar.2023.1185026

**Published:** 2023-08-14

**Authors:** Shuyan Li, Yan Ma, Hongling Sun, Zijun Ni, Shurong Hu, Yan Chen, Meijuan Lan

**Affiliations:** ^1^ Department of Nursing, The Second Affiliated Hospital, Zhejiang University School of Medicine, Hangzhou, China; ^2^ Center for Inflammatory Bowel Diseases, Department of Gastroenterology, The Second Affiliated Hospital, Zhejiang University School of Medicine, Hangzhou, China

**Keywords:** adherence, medication belief, Crohn’s disease, infliximab, China

## Abstract

**Objective:** Crohn’s disease (CD) is an incurable chronic disease that requires long-term treatment. As an anti-tumor necrosis factor (TNF) agent, Infliximab (IFX) is widely used in the treatment of Crohn’s disease, while the adherence is not high. The purpose of this study was to investigate the adherence to IFX among CD patients in China and evaluate the association between medication belief and IFX adherence.

**Methods:** Demographic data, clinical information and patients’ medication beliefs were collected using an online questionnaire and reviewing electronic medical records (EMRs). The Beliefs about Medicines Questionnaire (BMQ)-specific was used to assess medication beliefs which contains the BMQ-specific concern score and the BMQ-specific necessity score. An evaluation of adherence factors was conducted using univariate and multidimensional logistic regression analyses.

**Results:** In all, 166 CD patients responded the online questionnaire among which 77 (46.39%) patients had high adherence. The BMQ-specific concern score in patients in low adherence was 30.00 and in high adherence patients was 27.50, and patients with lower BMQ-specific concern score had higher adherence (*p* = 0.013). The multiple regression analysis showed that the BMQ-specific concern score (OR = 0.940, 95% CI: 0.888–0.996) significantly affected the IFX adherence in CD patients. Otherwise, gender, marital status, time spent on the way (including the waiting time in infusion center) and accommodation to the center were also the influencing factors of adherence.

**Conclusion:** The IFX adherence to CD in China was not high. Medicine concerns may be predictive factor of adherence. Education, the duration of IFX therapy and experience of adverse effects were not significantly associated with IFX adherence. By enhancing knowledge and relieving medicine concerns, we may increase patients’ adherence to IFX.

## Introduction

Crohn’s disease (CD) is a type of inflammatory bowel disease (IBD), which primarily affects the digestive system (Loftus, 2004). It is an incurable chronic disease that requires long-term treatment. Anti-inflammatory and immunosuppressive medications have become the standard of treatment for CD (Agrawal et al., 2020). Besides, biological agents have been widely used in the recent years and have greatly improved the remission rate in CD patients who were refractory to traditional medicine (Mastronardi et al., 2019). Anti-tumor necrosis factor (TNF) agents such as infliximab (IFX) is the most used biological agent, and has diminished hospitalizations and surgeries related to CD significantly (Lichtenstein et al., 2005).

Medication adherence is an important determinant of outcomes in patients with chronic diseases. The definition of adherence to medication is the degree to which a person’s behavior of taking medication, following a diet, and/or implementing lifestyle changes corresponds with their healthcare provider’s recommendations (Chew et al., 2020). Several reports have shown that 30%–50% of patients with chronic diseases have low medication adherence (Tamura et al., 2020) and a large meta-analysis examining medication adherence in IBD reported variable adherence rates, ranging from 7% to 72% (Jackson et al., 2010). Non-adherence to infliximab treatment increases the risk of treatment failure and developing immunogenicity to anti-TNF agents, which contributes to increasing healthcare cost (Kane and Shaya, 2008; Kane et al., 2009; Van Assche et al., 2010). Study showed that compared with adherent patients, the all-cause medical expenses and CD related medical expenses of non-adherent patients were 81% and 94% higher, respectively (Kane et al., 2009). A systematic review revealed that pooled adherence was 70.7% in infliximab-treated patients in IBD (11). Furthermore, females, smokers, and patients accompanied by psychiatric comorbidities are at increased risk of nonadherence (Fidder et al., 2013; Lopez et al., 2013). However, to our best knowledge, no study on adherence to infliximab among CD patients has been performed in China. A rapid increase in IBD has been observed in China over the past decade. It is expected that 1.5 million people in China will suffer from IBD by 2025 (Kaplan, 2015). IFX is the first anti-tumor necrosis factor agents approved for the treatment of CD patients in China. Therefore, it is necessary to investigate IFX adherence in China.

Among the predictors of medication adherence, the most significant one is medication belief (Mitzel and Vanable, 2020). Medication belief refers to an individual’s view of medication., which not only includes the cognitive responses to medication, but also includes the views on the harmfulness of the medication and maintenance therapy. Several studies have reported the significant correlation between medication adherence and medication belief (Horne et al., 2013; Chapman et al., 2014). To date, the study of medication belief has mainly focused on chronic disease, such as high blood pressure (Suarez-Arguello et al., 2022), ischemic stroke (Chen et al., 2019), diabetes (Mohammadi et al., 2018) and so on. A meta-analysis showed that if the patient has a higher belief in the necessity of taking medicine and a lower belief in the concern of harmfulness, the patient will show better adherence (Adem et al., 2021). In recent years, our research teams have focused on the medication adherence in IBD patients, we found medication belief is associated with improved adherence to exclusive enteral nutrition in patients with CD patients (Li et al., 2021). However, adherence to IFX among Chinese CD patients and its association with medication belief remain unclear.

In this study, we assessed adherence to IFX among patients with CD in China and evaluated the relationship between medication belief and IFX adherence.

## Methods

### Patient population and design

**FIGURE 1:**
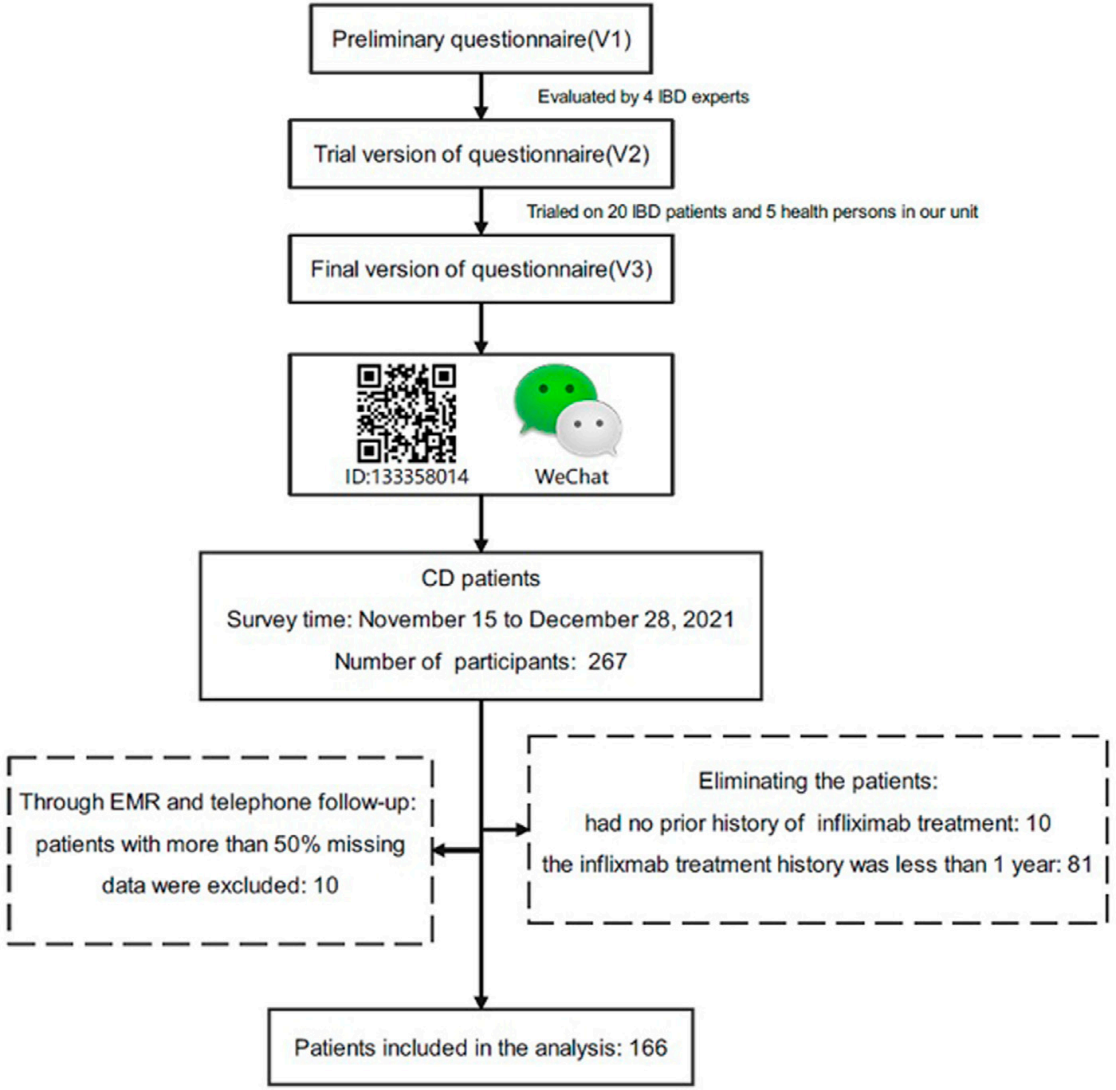
Flow chart of the study. The questionnaire was evaluated and modified by IBD experts and was produced by Wenjuanxing. We sent the questionnaire to the WeChat group for patient management and 267 patients responded, out of which 101 respondents were excluded.

The study was conducted at the Second Affiliated Hospital, Zhejiang University School of Medicine (SAHZU) from November 15 to 28 December 2021. All CD patients treated with infliximab were retrospectively identified in the electronic medical records (EMRs) from the SAHZU Crohn’s and Colitis Center. The inclusion criteria were as follows: patients with a confirmed diagnosis of CD; were treated with infliximab for at least 12 weeks; were informed consent. In our study, the infliximab was dissolved in saline and infused by intravenous infusion. IV infusion doses were at least 5 mg/kg. The same dose was given at 2 weeks, 6 weeks and every 8 weeks after the first administration. Infliximab regimen may be adjusted by the IBD specialists if the disease changes during the treatment. Nobody had a history of neurological or psychiatric conditions. As shown in Figure 1, we created an online questionnaire which was evaluated and modified by IBD experts and feedback from IBD patients after filling in. At the same time, healthy people were invited to complete the questionnaire and gave opinions on the readability of the questionnaire. And then we used the WeChat-based Questionnaire Star application generating a Quick Response code (OR code). Patients scanned the code and filled in the questionnaire. In addition, clinical information was gathered by reviewing EMRs and telephone follow-up. A total of 267 patients responded this online questionnaire, out of which 101 (37.83%) respondents had to be excluded: 10 patients had no prior history of infliximab treatment, 81 patients’ the medication history of infliximab were less than 1 year 10 patients had more than 50% missing data through EMRs and telephone follow-up were excluded.

### Clinical data collection

Three parts of data collection were conducted: Demographic data, clinical information and patients’ medication beliefs. These data were considered possible influencing factors on the results regarding non-adherence to infliximab treatment from previous studies (Lopez et al., 2013; van der Have et al., 2016; Govani et al., 2018). All the patients’ demographic data, including sex, age, marital status, education level, employment status, monthly income, smoking status and health insurance.

The clinical information was divided into two parts, disease related data and infliximab treatment data. Including disease duration, disease localization (was evaluated by Montreal classification), disease activity (was measured through Erythrocyte Sedimentation Rate (ESR), C-reactive protein (CRP), albumin and fecal calprotectin), family history, surgical history, concomitant therapy and complications. Infliximab treatment data including duration of infliximab, adverse effects and the convenience of medical care, for example, “What is your primary mode of transportation to the infusion center you visit most often? The total time (including waiting time) you spent to the infusion center? How far are you from your most frequent infusion center? What is the total cost of transportation to and from the infusion center you visit most often? If accommodation is required for the visit, what is the total approximate cost of accommodation?”

The Beliefs about Medicines Questionnaire (BMQ)-specific was used to assess medication beliefs (Horne et al., 1999). It has 10 items, 5 items on medication necessity, and 5 items on medication concern. A 5-point Likert scale was used for each item, ranging from 1 (strongly disagree) to 5 (strongly agree). The questionnaire has a score range of 5–25; higher scores indicate stronger levels of treatment beliefs. A patient’s overall belief about infliximab treatment can be derived by subtracting the average 5 item medication concerns scale score from their average 5 item medication necessity scale score, with a range of −20 to 20. Based on median scores, patients were categorized into High-BMQ and Low-BMQ.

### Adherence measures

Adherence was judged by comparing the theoretical infusion times with the actual infusion times in recent 1 year, which was referred to the definition of modified medication possession radio (mMPR) (Steiner TDK et al., 1988). Participants who adhered to their treatment plan at 90% or more were considered high adherence; those who adhered at less than 90% were considered low adherence (Ramos et al., 2022). If the infusion time was advanced or delayed for more than 1 week, it was considered as low adherence.

### Statistical analysis

All statistical analyses were performed using SPSS software (version 26.0, IBM Corp). Continuous variables were presented as means±standard deviations (SD) or means with interquartile range (IQR), and categorical variables were expressed as percentages. Student’s t-test and Chi-square test were used for statistical analysis of the data. Multivariate logistic regression analyses were performed to assess whether the individual variables were related to the adherence to Infliximab. In our analysis, *p* < 0.05 was considered significant. A multiple regression analysis was conducted with the adherence as the dependent variable. Factors with *p* < 0.2 in univariate analysis were included in multivariate analysis (using a forward-LR stepwise regression procedure).

### Ethical considerations

This study was reviewed and approved by the medical ethics committee of the Second Affiliated Hospital, Zhejiang University School of Medicine (No. 2021 0901).

## Results

### Patient inclusion and baseline characteristics

A total of 166 CD patients responded this online questionnaire. Of them, the median age was 32 years old, 115 (69.3%) patients were male, 107 (64.5%) had married; approximately 54.8% of the patients attained intermediate level of education (senior high or technical secondary school and college education); the median duration of disease was 4 years. In terms of adverse effects of infliximab, 61 (36.7%) had suffered or were currently suffering adverse effects (such as chest tightness, rash, difficulty breathing, pneumonia, tuberculosis and so on); The duration of infliximab use was from 2–4 years with a median of 3 years. Other characteristics were listed in Table 1.

**TABLE 1:** Baseline characteristics of respondents (n = 166).

Characteristics		n (%)
Age^a^	32.00 (25.00,39.00)	
Duration of disease (years)^a^	4.00 (2.00,6.00)	
Gender	Female	51 (30.7)
	Male	115 (69.3)
Marital status	Non-Married	59 (35.5)
	Married	107 (64.5)
Education ^b^	Low	53 (31.9)
	Intermediate	91 (54.8)
	High	22 (13.3)
Employment	No	59 (35.5)
	Yes	107 (64.5)
Family income(monthly)	<¥5000	35 (21.1)
	¥5001-¥10000	48 (28.9)
	¥10001-¥20000	49 (29.5)
	>¥20001	34 (20.5)
Smoking	Negative	135 (81.3)
	Active	31 (18.7)
Commercial insurance	No	124 (74.7)
	Yes	42 (25.3)
Montreal Classification	L1 (ileal localization)	63 (37.9)
	L2 (colonic localization)	7 (4.2)
	L3 (ileocolonic localization)	93 (56.0)
	L4a (from mouth to treitz ligament)	13 (7.8)
	L4b (from treitz ligament to distal 1/3 ileum)	65 (39.2)
	B1 (non-stricturing,non-penetrating behavior)	84 (50.6)
	B2 (stricturing behavior)	51 (30.7)
	B3 (penetrating behavior)	31 (18.8)
	p (perineal disease)	84 (50.6)
IBD-related surgery ^c^	No	83 (50.0)
	Yes	83 (50.0)
Combined medication	Amino salicylates	35 (21.1)
	Glucocorticoid	2 (1.2)
	Oral immunosuppressants	72 (43.4)
	Enteral nutrition	89 (20.5)
Adverse effects of infliximab ^d^	Never	105 (63.3)
	Have suffered or are currently suffering	61 (36.7)
Duration of infliximab^a^	3.00 (2.00,4.00)	
CRP(g/L)^a^	11.93 (0.90–15.63)	
ESR(mm/h)^a^	14.51 (3.75–21.00)	
ALB(g/L)^a^	40.81 (37.76–45.43)	
Fecal calprotectin(μg/g)^a^	1,001.03 (280.80–1800.00)	

^a^Presented as median(P_25_,P_75_).

^b^ Low level included junior high school education and below, intermediate level included senior high or technical secondary school and college education, and high level included bachelor or graduated education and above.

^c^ It was defined as any intestinal or perianal surgical procedure performed for underlying IBD.

^d^ Adverse effects of infliximab: such as chest tightness, rash, difficulty breathing, pneumonia, tuberculosis and so on.

### Associations between participants’ characteristics and adherence to infliximab

Only 77 (46.39%) patients had high adherence, while the other 89 (53.61%) had low adherence. A univariate analysis of clinical and demographic characteristics was conducted to explore the factors associated with adherence to infliximab. Patients with female gender, married status, and experience of adverse effects showed lower adherence. Also, the shorter time patients spent to the infusion (*p* < 0.001) and the lower cost of accommodation to the infusion center (*p* = 0.002), the higher adherence they reported (Table 2).

**TABLE 2:** Predictive Factors for Adherence to infliximab (Univariate Analysis).

Characteristics classification		Low adherence	High adherence	*p*-value
Age		30.00 (25.00,36.50)	33.00 (26.00,42.50)	0.143
Duration of disease		4.00 (2.50,6.00)	4.00 (2.00,6.00)	0.715
Gender	Female	21 (41.2)	30 (58.8)	0.032
	Male	68 (59.1)	47 (40.9)	
Marital status	Non-Married	40 (67.8)	19 (32.2)	0.007
	Married	49 (45.8)	58 (54.2)	
Education ^a^	Low	32 (60.4)	21 (39.6)	0.297
	Intermediate	48 (52.7)	43 (47.3)	
	High	9 (40.9)	13 (59.1)	
Employment	No	31 (52.5)	28 (47.5)	0.837
	Yes	58 (54.2)	49 (45.8)	
Family income(monthly)	≤¥10000	43 (51.8)	40 (48.2)	0.641
	>¥10000	46 (55.4)	37 (44.6)	
Smoking	Negative	70 (51.9)	65 (48.1)	0.342
	Active	19 (61.3)	12 (38.7)	
Commercial insurance	No	66 (53.2)	58 (46.8)	0.863
	Yes	23 (54.8)	19 (45.2)	
IBD-related surgery ^b^	No	45 (54.2)	38 (45.8)	0.876
	Yes	44 (53.0)	39 (47.0)	
Adverse effects of infliximab ^c^	Never	47 (44.8)	58 (55.2)	0.003
	Have suffered or are currently suffering	42 (68.9)	19 (31.1)	
Duration of infliximab		3.00 (2.00,4.00)	3.00 (2.00,4.00)	0.711
The total time patient spent to the infusion center every time				<0.001
Short time spent (<12 h)		64 (46.7)	73 (53.3)	
Long time spent (≥12 h)		25 (86.2)	4 (13.8)	
The distance from home to infusion center				0.372
Short distance (<10 km)		19 (47.5)	21 (52.5)	
Long distance (≥10 km)		70 (55.6)	56 (44.4)	
The total cost of transportation to and from the infusion center every time				0.334
Low transportation costs (<¥500)		84 (52.8)	75 (47.2)	
High transportation costs (≥¥500)		5 (71.4)	2 (28.6)	
The total cost of accommodation to the infusion center every time				0.002
Low accommodation (<¥100)		39 (42.9)	52 (57.1)	
High accommodation (≥¥100)		50 (66.7)	25 (33.3)	

^a^ Low level included junior high school education and below, intermediate level included senior high or technical secondary school and college education, and high level included bachelor or graduated education and above.

^b^ It was defined as any intestinal or perianal surgical procedure performed for underlying IBD.

^c^ Adverse effects of infliximab: such as chest tightness, rash, difficulty breathing, pneumonia, tuberculosis and so on.

### Influence of medication belief on adherence to infliximab

The BMQ-specific concern score, BMQ-specific necessity score and BMQ-specific score were showed in Table 3. The BMQ-specific concern score in patients with low adherence was higher than high adherence patients (30.00 vs. 27.50, *p* = 0.013). The BMQ-specific score in patients in low adherence was 00.00 and in high adherence patients was 5.00. Patients were divided into the high-BMQ group (BMQ-Specific score >0) and low-BMQ group (BMQ- Specific score ≤0). More patients with a high BMQ score had high adherence to IFX compared to those with a low BMQ score (*p* < 0.001).

**TABLE 3:** Influence of medication belief on adherence to infliximab.

Characteristics classification		Low adherence	High adherence	*p*-value
BMQ-specific concern		30.00 (25.00,33.75)	27.50 (20.00,32.50)	0.013
BMQ-specific necessity		32.50 (27.50,37.50)	32.50 (27.50,35.00)	0.723
BMQ-Specific		0.00 (0.00,6.25)	5.00 (0.00,10.00)	0.023
BMQ-Specific	Low-BMQ^a^	61 (71.8%)	24 (28.2%)	<0.001
	High-BMQ^b^	28 (34.6%)	53 (65.4%)	

^a^ Low-BMQ: the BMQ-Specific score ≤0 was defined as low-BMQ.

^b^ High-BMQ: the BMQ-Specific score>0 was defined as high-BMQ.

**FIGURE 2:**
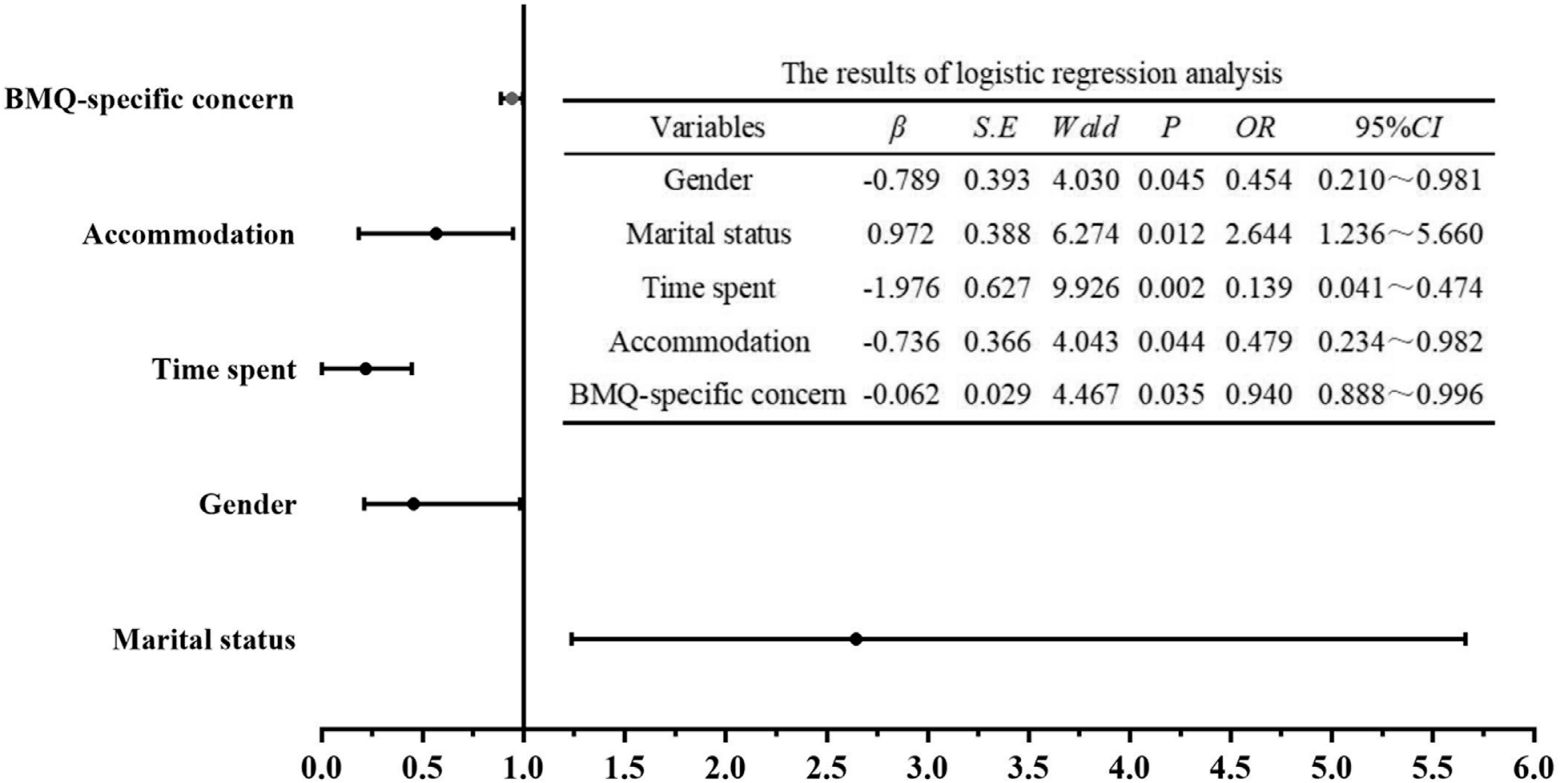
Multi-factor logistic regression models for medication adherence in IFX. The multiple regression analysis showed five variables, including BMQ-specific concern score, gender, marital status, time spent on the way and accommodation to the center were the main factors affecting the IFX adherence in CD patients.

The multiple regression analysis showed that the BMQ-specific concern score (OR = 0.940,95%CI:0.888–0.996) significantly affected the IFX adherence in CD patients. Other factors affecting adherence included gender (OR = 0.454,95%CI:0.210–0.981), marital status (OR = 0.454,95%CI:0.210–0.981), time spent on the way (OR = 0.139,95%CI:0.041–0.474), accommodation to the center (OR = 0.479,95%CI:0.234–0.982). The result was presented in Figure 2.

## Discussion

CD is a chronic disease in which required long-term pharmacotherapy and one of the key elements of medication efficacy is adherence. A patient’s adherence is measured by his or her ability to follow medical advice, such as taking medications and changing his or her lifestyle. Non-adherence of IBD may lead to treatment failure, increased hospitalization rate and treatment costs. However, the adherence to IFX therapy in Chinese CD patients was rarely reported. In our study we investigated adherence to IFX among CD patients and the factors associated with IFX adherence. We found almost more than half of IFX-treated CD patients were low adherent in China, which was varied crossed studies (Kitney JMT et al., 2009; Carter et al., 2011). For example, Carter et al. (2011) showed that the IFX adherent rate in CD patients was 57.1% (Carter et al., 2011); In pediatric IBD patients, Kitney’s study showed that the adherent rate for IFX use was 79.8% (Kitney JMT et al., 2009). Reasons for the differences in adherence could be attributable to differences in the methods of assessing medication adherence. In our study, high adherence was defined as an MPR higher than 90%, which applied a more exacting criterion by defining high adherence higher than 80% Carter et al. (2011). Participant adherence data relied on self-reported which may over-estimate. In addition, there may be differences in these results between countries with different health systems containing social and economic barriers that prevent medication adherence (Hu et al., 2020).

In our study, we found that adherence is influenced by gender, marital status, convenience (such as the total time patient spent to the infusion center every time and the total cost of accommodation to the infusion center every time). Compared to non-married patients, married patients had a higher adherence. There may be a greater level of socioeconomic status in married patients, as well as less emotional burden (Zhao et al., 2019). They could obtain emotional and financial support from their spouses or children (Feng et al., 2020). To ensure adherence and efficacy of treatment, the role of convenience may become even more significant (Centonze et al., 2021). IFX is administered intravenously. In China, patients need go to integrative hospital with a professional infusion department to receive IFX therapy. We found that the longer time the patient spent to the infusion center (including waiting time), the poorer medicine adherence they were, and the same is true for cost of accommodation There was higher adherence in the shorter time spent (<12 h) and lower accommodation cost (<¥100) in our study. One possible explanation was that their treatment process was burdened by the time and cost they had to spend on IFX infusion.

In this study, we found that BMQ-specific concern but not BMQ-specific necessity was significant associated factor for adherence. IFX is a chimeric monoclonal antibody against tumor necrosis factor alpha (TNF-α), and there are side effects associated with IFX treatment. The common side effects of IFX are serious infection, malignancies, infusion-related reaction and so on. Opportunistic infections are a major safety concern in patients with IBD, especially as IFX therapy becomes more widespread (Park et al., 2020). The most common opportunistic infection of IFX therapy is tuberculosis (TB) by destroying the granuloma integrity and increasing the reactivation of latent TB infection (Ford and Peyrin-Biroulet, 2013). Similarly, it can stimulate hepatitis B virus (HBV) resulting viral reactivation (Li et al., 2017b). In China, the prevalence of smear-positive tuberculosis, which approximates 59/100,000, is one of the highest in the world (Li et al., 2017a). And also infection with HBV is most prevalent in China (Dai et al., 2019). Therefore, the patient’s concern is truly a major clinical problem. We can improve adherence by relieving patients’ medication concerns. Clinicians should adequately explain to the patients the side effects of IFX, especially about opportunistic infections. There must be a balance between therapy and adverse effects. Screening the latent TB and HBV infection before and during IFX therapy for latent TB and HBV infection can ensure the safety of medications. Nurse can also play a critical role in helping patients to understand and take medication properly by bridging the gap between them and their practitioners (Jamison et al., 2017). They could help patients to improve their knowledge about IBD and understand the mode of action of IFX, and thus to improve the self-management abilities, self-health-promoting behaviors, and medical adherence. In addition, in order to ensure the safety of patients during medication administration, it is crucial to set up or update guidelines, to create a conducive environment, and to trained nurses on how to administer medications safely.

To our knowledge, this is the first study exploring the influence of medication belief on adherence to IFX in CD patients in China, and analyzing the adherence-influencing factors. Our findings reflected significant impacts of medicine concerns on IFX therapy adherence. There are also a few limitations in this study. Firstly, the study recruited patients from a single tertiary medical center, which may have led to selection bias. Secondly, our study only investigated the samples from CD patients, nevertheless, definitive studies should be conducted in patients with ulcerative colitis. Thirdly, previous studies have found psychosocial factors would affect adherence, such as anxiety and depression (Dolovich et al., 2021). In our study, these factors were not considered.

## Conclusion

In conclusion, the IFX adherence in Chinese CD patients was calling for improvement. Gender, marital status, convenience, medicine concerns may be predictive factors of adherence. Enhancing knowledge and relieving medicine concerns in CD patients may increase the treatment adherence on IFX.

## Data Availability

The raw data supporting the conclusions of this article will be made available by the authors, without undue reservation.
